# A Little Politics

**Published:** 1880-07

**Authors:** 


### A LITTLE POLITICS.

Here we are again, in the midst of another political campaign. Scarcely have we recovered from the excitement of one, ere we are plunged into the disquietude of another. We are kept too much agitated. Laboring people,—business men,—all, save the mischievous politicians, have their attention too much diverted from healthful, useful pursuits, to that of the ever busy and noisy politician.

It would seem, immediately that the President of the United States is seated in his executive chair, he calls about him—not the best advisors that the country can produce, but the cunningest and most influential politicians—men that are supposed to control conventions and large bodies of voters, to the end that a renomination and re-election may be secured

A President or any other officer of the Nation or State cannot be an honest one when so handicapped. He cannot serve his country according to his honest convictions of right, even if he would. He must obey the commands of party leaders, that the largest number of party men may be rewarded and retained in lucrative and often superfluous offices.

Every city and town has its prominent politicians, gentlemen of leisure, who have no visible means of support save that which is obtained by some mysterious means known only to those who "run the political machine." It seems to be the duty of these men to keep the political cauldron boiling, thereby fixing the attention of honestly busy men upon politics to the detriment of their own business and that of the community in general.—So it seems to a man —not "up a tree," but to one in the woods, away from the busy cares of men, calmly meditating upon the affairs of the outside world.

We are no politician. We never vote with a party, for the party's sake. We are not versed, therefore, in the reasonings and maneuverings of party men. But this is the remedy which occurs to us, for getting rid of these too frequently occurring political excitements which are certainly threatening the stability of our government.

Let us so amend the Constitution that we may elect our President, Congressmen and Governors for eight or ten years. Let us make them ineligible for office ever afterward, and retire them on half pay, so that they may live comfortably for the remainder of their days. An officer under this condition of affairs would have no incentive to do otherwise than serve his country faithfully. He could select his own counsellors without help or hindrance of the politicians, of whom he need ask no favors, and therefore be required to grant none.

We would then have less political excitement —less distraction from business pursuits—fewer professional politicians.

If the independent *press* of the country would present this matter to the people, we doubt not that in time, such a reform could be worked to the everlasting benefit of the nation.

